# Ultra-Responses of *Asphodelus tenuifolius* L. (Wild Onion) and *Convolvulus arvensis* L. (Field Bindweed) against Shoot Extract of *Trianthema portulacastrum* L. (Horse Purslane)

**DOI:** 10.3390/plants12030458

**Published:** 2023-01-19

**Authors:** Muhammad Shahid Hassan, Nargis Naz, Habib Ali, Basharat Ali, Muhammad Akram, Rashid Iqbal, Sidra Ajmal, Baber Ali, Sezai Ercisli, Kirill S. Golokhvast, Zeshan Hassan

**Affiliations:** 1 Department of Botany, The Islamia University of Bahawalpur, Bahawalpur 62100, Pakistan; 2 Department of Agricultural Engineering, Khawja Freed University of Engineering and Information Technology, Rahim Yar Khan 64200, Pakistan; 3 Department of Agronomy, Faculty of Agriculture and Environment, The Islamia University of Bahawalpur, Bahawalpur 63100, Pakistan; 4 Department of Plant Sciences, Quaid-i-Azam University, Islamabad 45320, Pakistan; 5 Department of Horticulture, Faculty of Agriculture, Ataturk University, 25240 Erzurum, Turkey; 6 Siberian Federal Scientific Center of Agrobiotechnology RAS, 2b Centralnaya, Presidium, 630501 Krasnoobsk, Russia; 7 College of Agriculture, Bahauddin Zakariya University, Multan, Bahadur Sub Campus, Layyah 31200, Pakistan

**Keywords:** allelochemicals, dry weight, root length, shoot extract, *Trianthema portulacastrum*, weeds

## Abstract

Weed infestation is a prime challenge coupled with lowering crop production owing to their competition with crop plants for available resources such as nutrients, water, space, moisture, and sunlight. Among weed control methods, the implementation of synthetic herbicides offers an instant solution for getting rid of weeds; however, they are a direct source of potential hazards for humans and generate resistance against synthetic weedicides, making them less effective. Allelopathy is something that happens in nature that can be used as a weed control method that increases crop yield and decreases dependency on synthetic chemicals. The mode of action of some phytochemicals corresponds to synthetic herbicides. Due to this feature, allelochemicals are used as bio-herbicides in weed management and prove more environmentally friendly than synthetic weedicides. The present investigation aims to assess the ultra-responses of *A. tenuifolius* and *C. arvensis*, while growing them in a pot experiment. Various levels of shoot extract (L2, L3, and L4) of *T. portulacastrum* along with the L1 (distilled water) and L5 (synthetic herbicide) were applied to the weeds. Results indicated that aqueous extracts of shoot of *T. portulacastrum* significantly (*p* ≤ 0.05) affect all the measured traits of weeds and their effects were concentration specific. All morphological parameters were suppressed due to biotic stress with an increase in free amino acids and calcium ions along with a decline in metaxylem cell area and cortical thickness in the root, while the vascular bundle area increased. The shoot extract intrusive with metabolisms corresponded with the synthetic herbicide. It is concluded that *Trianthema* shoot extract has a powerful phytotoxic impact on weeds (*A. tenuifolius* and *C. arvensis*) and can be used in bio-herbicide production.

## 1. Introduction

Weed profusion limits the utilization of water and nutrients in crop plants due to their competitive aptitude. Weed infestation is a major challenge associated with lowering crop production because they also struggle for available resources like space, moisture, and sunlight [1]. Moreover, weeds influence the crops by discharging allelochemicals, which affect the crops by reducing growth. Consequently, about one-third of the yield loss of leading crops grown across the world is caused by weeds, while in Pakistan 11.5% of expected crop yield losses are due to weeds [2]. Among weed control methods, synthetic herbicides offer immediate and quick relief to the former in weed controlling; however, they are a direct source of potential hazards for humans and generate resistance against synthetic weedicides, making them less effective [3]. An innovative approach to alleviating the pessimistic impacts of synthetic herbicides on crop yield is by employing natural herbicides [4]. The massive challenging technique for a safer environment by applying these approaches, which are cheaper and more eco-friendly, for weed control has intensified exploration studies on crops and weed allelopathy. The herbicidal potential of plants that can produce allelochemicals is considered a major source of biological control and as the weeds develop resistance to synthetic chemical compounds, the significance and investigation of novel molecules are augmented. Hence, global researchers pay further attention to finding out some natural alternatives and biological control to decrease or curtail the dependence on synthetic herbicides. Allelopathy can be regarded as an efficient natural substitute for synthetic herbicides. Practically, it is observed that allelopathic plants liberate the active molecules into the environment, which exerts an effect on neighboring plants [5].

These allelochemicals are derivative metabolites that are synthesized in different metabolic activities and released in the environment by various parts (leaves, flowers, roots, seeds, and stems), by decomposition, or by leaching from plant residues [6,7]. Plants operate allelochemicals for their communication system between plant–plant, plant–insect, or plant–herbivores [8]. Allelopathy has been reported 2000 years ago and this phenomenon was observed as early as 300 B.C. by Theophrastus, who perceived the adverse effects of cabbage on a vine plant. The term allelopathy was applied by German plant physiologist Hans Molisch (1937) to describe the harmful effect of one plant upon the other [9]. Owing to the discrete nature of allelochemicals, they cannot be expected to demolish all types of weeds in agricultural fields. Therefore, it could function as a key factor of an integrated approach to weed management. Its advantages are its complexity, its enormous capacity for the devastation of weeds, and the minimal risk of environmental contamination. Allelochemical communication need further comprehensive laboratory study in an attempt to provide demand and give chances for the practical appliance of allelopathy in weed control to minimize the use of chemicals. These natural allelochemicals pose a negligible threat to the surroundings as compared to synthetic chemical compounds; this is the purpose behind developing natural chemicals, which may replace conventional pesticides [10].

*A. tenuifolius* (wild onion) is found as a weed in many winter crops but copiously infested the wheat and chickpea crops while *C. arvensis* (field bindweed) is a noxious weed infested in 32 cultivated crops [11] and it is a strong competitor due to a better-penetrated root system. It can tolerate a broad range of environmental circumstances in all types of soil. Moreover, this weed asphyxiates baby seedlings, grows quickly, and assaults the crops. Crop yield losses due to *C. arvensis* are about 20–70% and generate intricacy in harvesting [12]. *T. portulacastrum* is an important medicinal weed and is used for fever, jaundice dropsy, liver, and kidney diseases. It is also used as a vegetable due to its high nutritional value [13]. *T. portulacastrum* leaves and stems (shoot) contain various active substances such as p-hydroxybenzoic acid, vanillic acid, caffeic acid, ferulic acid, o-coumaric acid, protocatechuic acid, pyrogallic acid and trans-cinnamic acid [14]. Many researchers worked out the allelopathic potential of *T. portulacastrum*, but the use of an allelopathic extract of *T. portulacastrum* on *A. tenuifolius* and *C. arvensis* is the novelty of the present research. Accordingly, the current study aimed to analyze the allelopathic effect of *T. portulacastrum* shoot extract on *A. tenuifolius* and *C. arvensis.* For this motive, various morphological, physiological, and anatomical responses were sought.

## 2. Results

### 2.1. Morphological Characteristics

The results (Table 1) illustrated that there was a significant decline in all morphological characteristics of *A. tenuifolius* and *C. arvensis* by increasing allelopathic levels of shoot extract of *T. portulacastrum*. Root length, the number of leaves, root dry weight, and shoot dry weight reduced on increasing the level of shoot extract in *A. tenuifolius*; however, shoot length decreased gradually at all levels except at L3 (60%) and L4 (100%), in which the minimum and same reduction value was noticed. Similarly, shoot length, leaf area, shoot dry weight, root dry weight, and root length were reduced with an enhancement in the level of shoot extract in *C. arvensis*, but this decreasing trend was not ongoing at L4 (100%). However, herbicide treatment caused greater decline than the shoot extract in all morphological parameters of both weeds.

### 2.2. Physiological Characteristics

Free amino acid content increased significantly at all levels of shoot extract in *A. tenuifolius*, while there was a non-significant increase in this parameter in *C. arvensis.* A concentrated level of shoot extract caused a greater increase in *A. tenuifolius*, while in *C. arvensis*, diluted levels raised the content of free amino acids. Calcium ions gradually increased with increasing levels of shoot extract in *A. tenuifolius*, but a greater increase in this parameter was calculated at diluted levels (L2 and L3). Likewise, the herbicide treatment also caused an increase in both parameters in both weeds, but a slight reduction was recorded in calcium ions in *C. arvensis* (Table 1).

### 2.3. Anatomical Characteristics

(i) Root anatomy.

**Figure 1:**
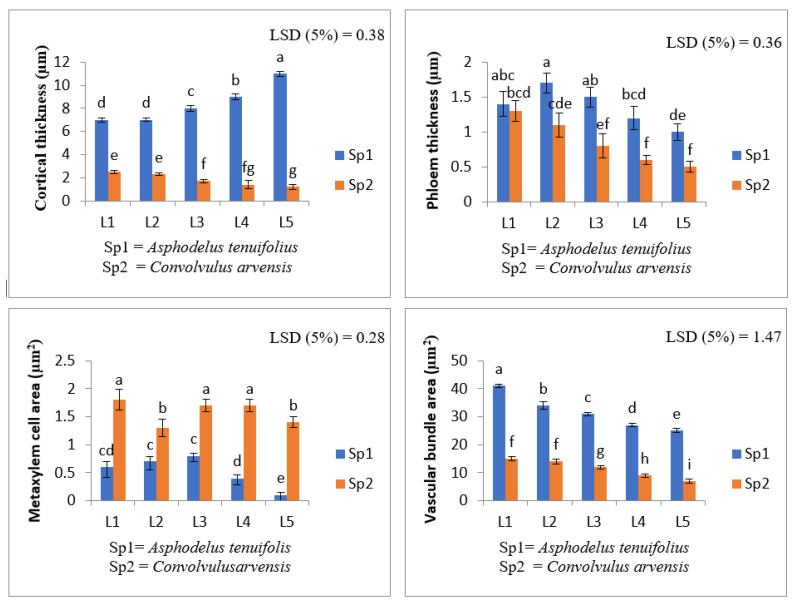
Effect of shoot extract of *T. portulacastrum* and herbicide on root anatomical parameters of *A. tenuifolius* and *C. arvensis.* L1 = distilled water; L2 = 30%; L3 = 60%; L4 = 100% and L5 = herbicide treatment. Means followed by the same letter did not significantly differ at *p* ≤ 0.05 according to LSD test.

**Figure 2:**
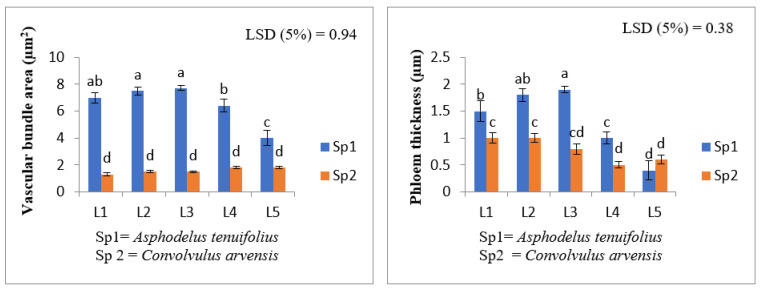
Effect of shoot extract of *T. portulacastrum* and herbicide on leaf anatomical parameters of *A. tenuifolius* and *C. arvensis.* L1 = distilled water; L2 = 30%; T3 = 60%; T4 = 100%; and T5 = herbicide treatment. Means followed by the same letter did not significantly differ at *p* ≤ 0.05 according to LSD test.

The cortical thickness in *A. tenuifolius* indicated a significant gradual increase with increasing levels of shoot extracts of *T. portulacastrum*. The herbicide level also caused an increase in this parameter. However, the decreasing trend was examined in *C. arvensis,* and a greater reduction was recorded in the more concentrated extract level. The herbicide also decreased the cortical thickness in *C. arvensis*. Phloem thickness in *A. tenuifolius* treated with shoot extract of *T. portulacastrum* displayed a reduction only at the L4 level, similar to the herbicide treatment (L5), but all other levels caused a slight increase in this parameter. Meanwhile, a declining trend was noticed at all shoot extract levels (Figure 1). The metaxylem cell area in *A. tenuifolius* treated with shoot water extract indicated an abrupt increasing trend at L2 and L3, while a reduction in this parameter was observed at L4 and L5 levels. A similar reduction was recorded at all levels of shoot extracts and herbicide treatment in *C. arvensis* in the vascular bundle area. The vascular bundle area treated with shoot water extract indicated a significant slight reduction at all levels in *A. tenuifolius* and *C. arvensis*. Meanwhile, the herbicide treatment caused the maximum decrease in vascular bundle area in both weed species (Figure 2).

(ii) Leaf anatomy.

**Figure 3:**
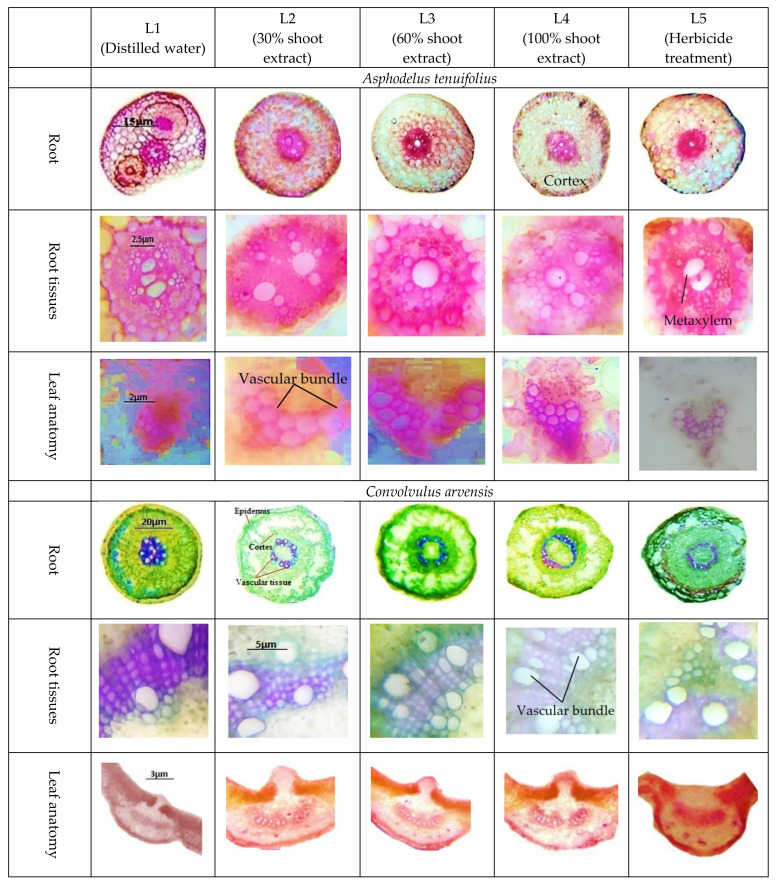
Root and leaf anatomy of *A. tenuifolius* and *C. arvensis* with applying different levels of shoot extract of *T. portulacastrum* and herbicide.

The vascular bundle area was increased significantly at all levels of shoot extract in *C. arvensis.* The maximum increase was recorded at a concentrated level (L4). Meanwhile, this increase was also reported in *A. tenuifolius*, while the maximum increase was noticed at a diluted level. However, at L4 and L5 levels in *A. tenuifolius,* there was a slight decline in this parameter (Figure 3).

## 3. Discussion

Plants face many harsh environmental stresses that impair their growth, development, metabolic processes, and production [15]. Various biotic and abiotic stresses are accountable for production losses in plants [16]. Weeds are biotic stress that causes major yield losses in crops. In developing countries, weeds are one of the chief biotic stresses to crop plants [17]. Weeds have been proven as the most noteworthy crop loss factor economically and environmentally. Diverse approaches to weed control comprise mechanical, chemical, and biological techniques. A widely used method is chemical weed control, which is unsustainable and improper for the environment and health of living organisms. However, a safe, budget-efficient, and environmentally friendly technique of weed regulation is biological control [18]. Currently, a potential substitute for weed control is allelopathy [19]. In allelopathy, plants produce biochemical compounds that hamper the growth, development, and reproduction of other plants [8]. Allelopathy has been proven a cost-effective and environmentally friendly weed controlling method [20].

The application of the highest concentration of shoot extract of *T. portulacastrum* in the present work illustrated a detrimental effect on the growth of the weeds. The root length as well as the shoot length was reduced in the present work. Analogous to the present results, the shoot and root length of various weeds (*Amaranthus graecizans, Amaranthus hybridus, Brachiaria reptans, E. colona, Hibiscus trionum, Portulaca oleraceae,* and *Setaria pumila*) were decreased [21]. The root and shoot of *Sorghum bicolor* and *Helianthus annuus* water extracts greatly suppressed both root and shoot length of the *Digera arvensis* and *C. arvensis* [22]. Corresponding outcomes were also described previously [20], who calculated that the extract of *T. portulacastrum* repressed the shoot length in *C. arvensis* significantly at the maturity stage. The gradual reduction in shoot length of *A. tenuifolius* and *C. arvensis* weed plants was recorded in the present research that was analogous to study of the Sutradhar et al. [23], who depicted that the shoot length decreased in jute plant along with the increasing level of extract of *T. portulacastrum*. Present results are also parallel with Hussain et al. [4], who described that *Acacia* phyllodes extract caused 50.78% inhibition in the shoot length of *Lactuca sativa*. Inhibition in shoot length may be coupled with the existence of certain allelopathic compounds with phytotoxic effects [23]. Malfunctioning in DNA replication, disturbance in mitochondrial reactions, and failure in cell division are accountable for shoot length reduction [24]. Several studies have perceived that many secondary metabolites like terpenoids, phenolics, and alkaloids and their derivatives are potent inhibitors for the dry weight of plants [25].

The reduction of root and shoot dry weight in the current work is in accordance with Ghimire et al. [26], who studied allelochemicals of *Miscanthus sacchariflorus* that reduced the dry weight of weed plants (*Oenothera biennis, Digitaria ciliaris, Chenopodium album, Artemisia princeps, Commelina communis*, and *Erigeron canadensis*). Naeem et al. [27] reported the dry mass reduction in *C. album* and *C. didymus* with an application of combined effects of sorghum + sunflower and sorghum + sunflower + mulberry extracts. The dry biomass of selected weeds was also restrained with the treatment of the synthetic herbicide. This decrease in dry weight may be linked with a reduction in enzyme activity as a result of a malfunctioning in the biosynthesis of materials. The shoot aqueous extract of *T. portulacastrum* decreased the leaf area and the number of leaves in tested weed plants. Zohaib et al. [28] studied that *M. parviflora* and *C. murale* aqueous extracts of different levels posed a negative effect on a number of leaves of barley. Al-Johani et al. [29] also confirmed that *M. parviflora* and *C. murale* aqueous extracts of various concentrations caused a negative effect on the leaf area of barley. Present outcomes are also analogous to the result of Sarabi et al. [30], who explored that *C. album* extract considerably declined the leaf area of *Zea mays*. Vaishali and Chturvedi [31] reported a diminution in the area of leaf in *M. capitata* by the appliance of leaf extracts of castor bean and chaste. This decline might be because of the existence of phytochemicals that impose inhibition in the synthesis of growth regulators like auxins, gibberellins, and other growth hormones resulting in an impairment in metabolism.

A high level of free amino acids is presumed to be the elevating protein content, which is due to a high decline in the protease activity during stress that is very significant for the hydrolysis of reserve proteins [32]. Analogous to our work, Maqbool et al. [33] illustrated that biotic stress enhances the level of free amino acids in corn plants. Corn plants under high abiotic stress revealed surges in metabolites of amino acids e.g., threonine, tryptophan, glutamate, myoinositol, beta-alanine, proline, and serine [34]. Frequently increased levels of calcium ions in the present work act as a key role in conserving the variety of structure and function of plant plasma membranes, ion control, maintaining cell wall structures, and control behavior of ion-exchange behavior and actions of cell wall enzymes [35]. Ca^2+^ signaling is a versatile mechanism that is common to an increasingly considerable plant nutrient and ion sensing as well as various adaptation processes. Xiong et al. [36] also perceived that abiotic stress was enough to cause an elevation of Ca^2+^ in plant leaves.

Plant roots being the direct connection with the soil water can be the prime sites of destruction or defensive line, and these are the first structures that can face any type of soil stress (Rewald et al. [37]. On the other hand, the cortical portion is the first type of tissue to experience soil stress, after the epidermis which is the outermost layer of cells. Modifications in anatomical structure happen in plants to cope with these conditions and support the plants to acquire adaptations for these challenging situations (Lamalakshmi et al. [38]. The present study indicated some anatomical changes under different levels of shoot extracts as well as at herbicide treatment. A decrease in cortical thickness in *C. arvensis* root with shoot extract was recorded in the present study. The cortical region reduction is the consequence of a decrease in the plant storage capacity and increases the sensitivity level of plants, which may cause severe tissue damage (Rahman et al. [39]. Metaxylem cell area decreased at concentrated treatments of shoot extract in *C. arvensis* and *A. tenuifolius*. As reported by many researchers, such as Hameed et al. [40], a decrease in metaxylem area in wheat varieties is observed at a higher level of leaf extract of *A. scholaris.* However, an increase in the metaxylem cell area of *A. tenuifolius* at diluted shoot extract was consistent with the finding of Chen et al. [41], who reported an increase in the metaxylem cell area and vascular bundle area at high salinity levels in wild barley.

Anatomical leaf modifications are the indication signals for various types of stresses in plants [42]. An increase in the vascular bundle area of the leaf at various levels of treatment is reinforced by Naz et al. [43], who elucidated the increase in vascular bundle area with increasing salinity stress levels. An increase in metaxylem cell area in *C. arvensis* at concentrated shoots extract levels favors the stronger tolerance against the biotic stresses, as water and mineral translocation may be easier, and this was a crucial adaptation against harsh environments (Ruiz et al. [44]). While the decrease in this parameter in *A. tenuifolius* was fully reinforced by Hameed et al. [40], who described that leaf extract of *A. scholaris* caused a decline in the metaxylem cell area in wheat lines. Vascular bundle area reduction in *A. tenuifolius* was supported by Nassar et al. [45], who affirmed the reduction in a vascular bundle area with the application of abiotic stress. This decline was helpful in the slighter deposition of water in the growth zone during stress conditions [46].

## 4. Materials and Methods

### 4.1. Anthology of Allelopathic Plant and Preparation of Extract

Fresh samples of *T. portulacastrum* were collected from the cotton fields during the summer season. The entire plants were rinsed rigorously and thoroughly with distilled water to remove the dust on the leaves and soil particles from the roots. Shoots were alienated from the roots and kept under shade for drying. Thereafter, the dried samples of shoots were crushed and ground, with the help of a grinder, into a fine powder and placed at room temperature in clean and sealed glass containers. The water extract was prepared by soaking 10 g of powder of shoots of *T. portulacastrum* in 100 mL distilled water (10% *w/v*) for 24 h at 20 °C. The extract was filtered with Whatman No.1 filter paper. The filtered solution was placed in the refrigerator as a stock solution. This extract solution made use of final concentrations of 30%, 60%, and 100% since we have figured out their concentration based on our pilot study where high concentrations caused more inhibition (data not shown).

### 4.2. Shoot Extract Levels Applied to Weeds

L1 = Control treatment (Distilled water)
L2 = 30% allelopathic aqueous treatment of Shoot
L3 = 60% allelopathic aqueous treatment of Shoot
L4 = 100% allelopathic aqueous treatment of Shoot
L5 = Herbicide treatment

### 4.3. Soil Analysis

The soil that was used in pots was analyzed for physicochemical characteristics such as pH (8.1) and electrical conductivity (1.97 ds/m) by pH/EC meter (WTW series InoLab pH/Cond 720). Available K (113 ppm), P (6 ppm), and organic matter (0.51%) along with texture (sandy loam) were determined according to Handbook No. 60 (USDA Laboratory Staff, 1954).

### 4.4. Collection of Seeds of Weeds and Conduction of Pot Experiment

**Figure 4:**
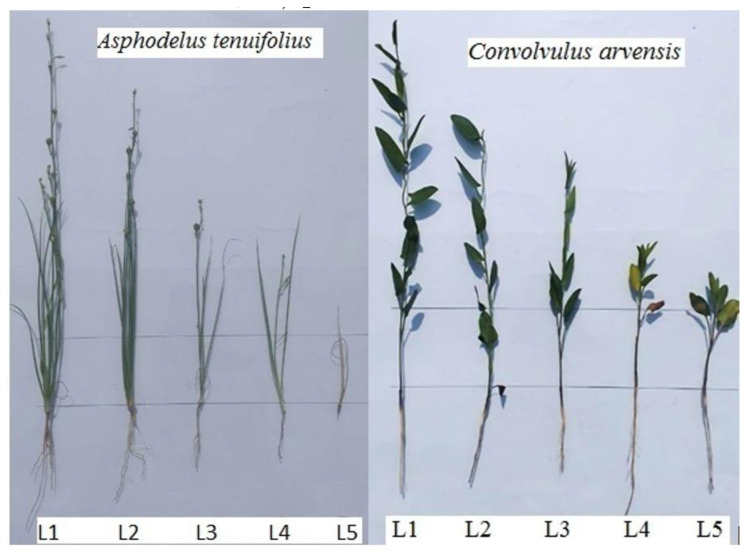
Effect of shoot water extract levels of *Trianthema portulacastrum* and herbicide (L1 = distilled water; L2 = 30%; L3 = 60%; L4 = 100%; and L5 = herbicide treatment) on the vegetative growth of *A. tenuifolius* and *C. arvensis*.

The seeds of *A. tenuifolius* and *C. arvensis* were collected at the maturity stage in April 2017 from the wheat field. A pot trial was performed in a complete randomized design at the botanical garden of the Islamia University of Bahawalpur, Punjab, Pakistan (located at 29.3783° N and 71.7647° E). From selected seeds of two weeds, after imbibition of seeds in cold water for 48 h, during December 2017, 15 seeds of each weed were sown in each pot having 5 kg of garden soil and this procedure was then repeated in 2018. The pots were regularly irrigated with tap water to maintain the moisture of the soil. After the germination had been completed, thinning was completed and ten healthy seedlings were left in each pot. The different specie’s pots were arranged into 5 groups with 3 replicates. L1 L2, L3, L4, and L5 groups (Figure 4) of pots were irrigated with distilled water, 30% shoot extract, 60% shoot extract, 100% shoot extract, and herbicide solution (Metafin Super 28.6% WDG), respectively, after 60 days of sowing seeds in pots [47].

### 4.5. Morphological Parameters

Plant samples were collected after the ten days of single treatment application at the vegetative stage to study their root length (cm), root dry weight (g/plant), shoot length (cm), shoot dry weight (g/plant), leaf area (cm^2^) and the number of leaves (per plant).

### 4.6. Physiological Parameters

The following physiological characteristics were studied from the samples collected at the vegetative stage:

(i) Free amino acids (mg/g d.wt.).

Free amino acid content was determined by Hamilton and Van Slyke [48]. The sample mixture was prepared with 1 cm^3^ of 10% pyridine and 1 cm^3^ of 2% ninhydrin solution in the supernatant. The optical density of the sample solution was noted at 570 nm by spectrophotometer.

(ii) Determination of Ca^2+^ (mg/g d.wt.).

The content of calcium ions was calculated by a flame photometer (Jenway, PFP-7, Göteborg, Sweden) according to Kacar [49]. For the determination of the ions, the sample of leaves was dried at 70 °C for 48 h. The calcium ion values with a flame photometer were determined by comparison with standard curves and the total amount was calculated.

### 4.7. Anatomical Parameters

For compound microscopic examination, the root and leaf specimens of two weeds such as *Asphodelus tenuifolius* and *Convolvulus arvensis* were prepared by killing and fixing in F.A.A. (10 mL formalin, 5 mL acetic acid, and 35 mL ethyl alcohol 95%); afterward, the samples were washed with distilled water and put into labeled bottles for further treatment. Freehand sectioning slides of various parts of selected plant species were prepared, then they underwent double-stained dehydration with safranin and fast green; afterward, they were cleared in xylene, and mounted in Canada balsam, and various cells and tissues of roots and leaves were examined. Measurements and photographs were taken using an ocular micrometer and a digital camera. Cortical thickness (µm), phloem thickness (µm), metaxylem cell area (µm^2^), and vascular bundle area (µm^2^) of root and cortical thickness (µm), phloem thickness (µm), metaxylem cell area (µm^2^), and vascular bundle area (µm^2^) of leaf parameters were calculated.

### 4.8. Statistical Analysis

The pot experiment was laid out in a completely randomized design (CRD) with three replicates. The collected data were subjected to the analysis of variance (ANOVA) and the LSD test was performed [50] using the software “Statistix version 8.1”.

## 5. Conclusions

Various weeds infest crops and impose drastic negative phytotoxic effects and *A. tenuifolius* and *C. arvensis* are the most challenging weeds of winter crops. The current work on the allelopathic effect of *T. portulacastrum* on *A. tenuifolius* and *C. arvensis* revealed that the shoot extract of *T. portulacastrum* can induce a significant inhibitory action on the growth of these two weeds by inducing alterations in the internal tissues. Without the introduction of new herbicide mechanisms, we could not control herbicide resistance, so allelopathy technology is getting popularity due to its safe and harmless nature. Results indicated that shoot extract works parallel to a synthetic herbicide. Therefore, *T. portulacastrum* shoot extract has a strong phytotoxic effect on weeds and can serve as bio-herbicide production.

## Figures and Tables

**Table 1:** ANOVA for the influence of shoot extract of *Trianthema portulacastrum* and herbicide on morphological and physiological parameters of *Asphodelus tenuifolius* and *Convolvulus arvensis* at the vegetative stage.

Treatment Levels	L1	L2	L3	L4	L5	LSD	CV	GM	F Value
Morphological parameters	*Asphodelus tenuifolius*								
RL (cm)	6.3 a	6 a	5 b	4 c	2 d	0.42	4.99	4.6	168 **
SL (cm)	21 a	20 a	20 a	18 b	10 c	1.19	3.68	17.8	142 **
RDW (g)	0.12 a	0.08 b	0.05 c	0.05 c	0.04 c	0.03	24.25	0.07	12.4 **
SDW (g)	2.2 a	1.7 b	1.5 b	1.0 c	0.6 d	0.32	12.78	1.4	36.1 **
NL (per plant)	13 a	12 a	8 b	4 c	3 c	3.82	26.22	8.0	14.0 **
LA (cm^2^)	10 a	7 b	4 c	4 c	3 c	1.15	11.27	5.6	62.6 **
Morphological parameters	*Convolvulus arvensis*								
RL (cm)	14 a	12 b	11 c	7 d	6 e	0.77	4.22	10	193 **
SL (cm)	19 a	15 b	12 c	9 d	7 e	1.18	5.25	12.4	161 **
RDW (g)	0.49 a	0.41 a	0.29 b	0.21 b	0.1 c	0.10	18.52	0.30	22.9 **
SDW (g)	1.0 a	0.7 b	0.4 c	0.3 c	0.2 c	0.27	29.16	0.52	14 **
NL (per plant)	14 a	10 b	7 bc	7 bc	5 c	3.04	19.46	8.6	13.2 **
LA (cm^2^)	4.2 a	3.0 b	2.5 bc	2.3 bc	1.5 c	1.04	21.15	2.7	9.16 **
Physiological parameters	*Asphodelus tenuifolius*								
TFA (mg/g)	7 c	8.6 b	8.6 b	9.9 a	8.3 b	0.41	2.65	8.5	63.4 **
Ca ions (mg/g)	5 c	7.4 a	8 a	8 a	6 b	0.77	6.17	6.9	29.5 **
Physiological parameters	*Convolvulus arvensis*								
TFA (mg/g)	6 b	7.3 a	7.8 a	7 ab	6.8 ab	1.23	9.7	7.0	2.9 ^NS^
Ca ions (mg/g)	6.5 b	7.5 a	7.5 a	6.5 b	6.3 b	0.64	5.17	6.8	8.2 **

L1 = distilled water; L2 = 30%; L3 = 60%; L4 = 100%; L5 = herbicide treatment; RL = root length; RDW= root dry weight; SL = shoot length; SDW = shoot dry weight; NL = number of leaves; LA = leaf area; TFA = total free amino acids; Ca = calcium ions; LSD = least significance difference; CV = coefficient variation; GM = grand mean; ** = significant at *p* ≤ 0.01; ^NS^ = non-significant. Means followed by the same letter did not significantly differ at *p* ≤ 0.05 according to LSD test.

## Data Availability

Data available on request.
